# Spatiotemporal mode-locking and dissipative solitons in multimode fiber lasers

**DOI:** 10.1038/s41377-023-01305-0

**Published:** 2023-10-30

**Authors:** Bo Cao, Chenxin Gao, Kewei Liu, Xiaosheng Xiao, Changxi Yang, Chengying Bao

**Affiliations:** 1 State Key Laboratory of Precision Measurement Technology and Instruments, Department of Precision Instruments, Tsinghua University, Beijing, 100084 China; 2 State Key Laboratory of Information Photonics and Optical Communications, School of Electronic Engineering, Beijing University of Posts and Telecommunications, Beijing, 100876 China

**Keywords:** Fibre lasers, Nonlinear optics

## Abstract

Multimode fiber (MMF) lasers are emerging as a remarkable testbed to study nonlinear spatiotemporal physics with potential applications spanning from high energy pulse generation, precision measurement to nonlinear microscopy. The underlying mechanism for the generation of ultrashort pulses, which can be understood as a spatiotempoal dissipative soliton (STDS), in the nonlinear multimode resonators is the spatiotemporal mode-locking (STML) with simultaneous synchronization of temporal and spatial modes. In this review, we first introduce the general principles of STML, with an emphasize on the STML dynamics with large intermode dispersion. Then, we present the recent progress of STML, including measurement techniques for STML, exotic nonlinear dynamics of STDS, and mode field engineering in MMF lasers. We conclude by outlining some perspectives that may advance STML in the near future.

## Abstract (web summary)

Spatiotemporal mode-locking in multimode fiber lasers: towards spatiotemporal dissipative solitons.

## Graphical abstract

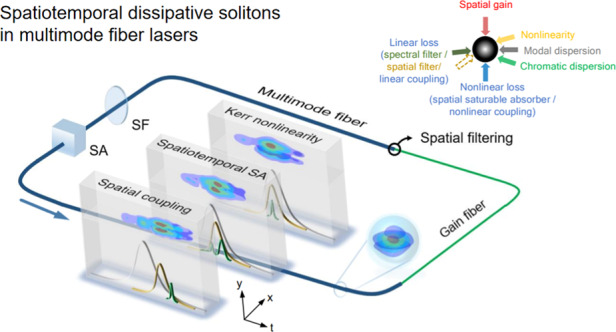

## Introduction

**Fig. 1:**
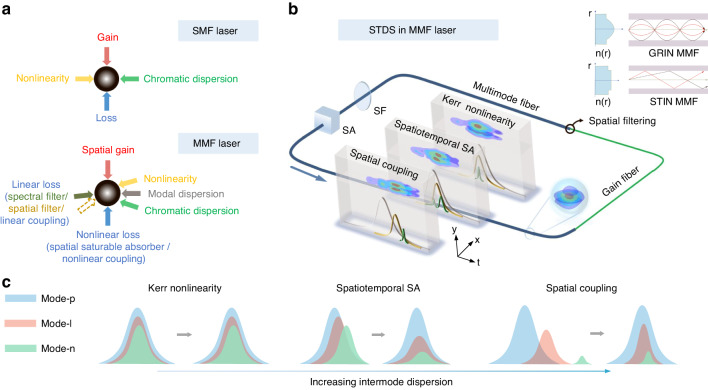
Concept of spatiotemporal mode-locking (STML) and spatiotemporal dissipative solitons (STDSs) in multimode fiber (MMF) lasers. **a** Schematic diagram of dissipative soliton formation in SMF lasers and MMF lasers. Dissipative soliton generation requires double balance between dispersion and nonlinearity as well as gain and loss. For a SMF laser, the dispersion corresponds to chromatic dispersion. In MMF lasers, intermode dispersion constitutes a new dimension that needs to be balanced for STDS formation. Steady propagation of STDSs in MMF lasers needs delicate balance between gain, nonlinear loss, linear loss, nonlinearity and dispersion. **b** An illustration of a typical STML MMF laser layout. Depending on the value of intermode dispersion, different mechanisms (Kerr nonlinearity, spatiotemporal saturable absorber, spatial coupling/filtering) can contribute differently to balancing the intermode dispersion. The three body diagrams show the snapshot of the 3D pulses and modal pulses (three curves in the diagram) after one round trip propagation. When the intermode dispersion is weak, Kerr nonlinearity can contribute to binding the modal pulse; when the intermode dispersion is large, modal pulses walk off, and saturable absorber (SA) and spatial coupling contribute significantly to counteracting the walk-off. SF, spectral filter. The inset shows an illustration for refractive index distribution and light propagation in a graded-index (GRIN) MMF and a step-index (STIN) MMF. **c** Schematic diagrams of pulse reactions for three mechanisms as intermode dispersion increases. Left: pulses for different spatial modes can bind together through Kerr nonlinearity under weak intermode dispersion. Middle: pulses slightly walk off under intermediate intermode dispersion. Spatiotemporal SA can reset the walk-off by saturable absorbing weak modes and redistributing energy among modes. Right: pulses walk off significantly under large intermode dispersion. Spatial coupling can attenuate weak modal pulses and redistributing energy from the mother-mode to the child-modes and alignes the modal pulse timing (see Section “STDS under large intermode dispersion”)

Solitons are self-sustained particle-like waves that widely exist in nonlinear systems. The inevitable dissipation in real systems leads to the concept of dissipative solitons which rely upon double balance between dispersion and nonlinearity as well as gain and loss (Fig. 1a)^1^. Optical systems are an excellent testbed to understand soliton dynamics^2–15^. The formation of temporal dissipative solitons in single-mode fiber (SMF) mode-locked lasers, which lay the foundation of modern ultrafast fiber lasers^6^, is achieved by synchronization of the longitudinal modes (temporal modes). Simultaneous synchronization of the transverse modes (spatial modes) and the longitudinal modes (temporal modes), i.e., spatiotemporal mode-locking (STML) is receiving growing interests in recent years^16–23^. Multimode fiber (MMF) lasers constitute a flexible platform to realize STML and spatiotemporal dissipative solitons (STDS, dissipative solitons circulating in nonlinear resonators with dynamics impacted by the spatial dimension).

MMFs were first introduced in 1970s in the very early development of fiber optics. However, they were not widely used for data transmission due to the large intermode dispersion. Recently, they have regained research interests as a possible solution to mode-multiplexed data transmission^24,25^, as well as a platform for nonlinear optics studies^26^. Many phenomena including spatiotemporal instability^27–29^, beam self-cleaning^30–35^, conservative multimode solitons^36–39^, soliton self-frequency shift^40^, supercontinuum generation^26,35,41–44^, intermode four-wave mixing^45–48^, control of nonlinear multimode interactions^49^ have been demonstrated in MMFs.

MMF lasers can also be used to realize STML and STDSs, which require delicate balance among linear and nonlinear effects, as shown in Fig. 1a. In fact, the feasibility of STML in multi-mode lasers has been proposed as early as the birth of lasers^50–52^. Nevertheless, it is only until 2017 that the first spatiotemporally mode-locked MMF laser was reported^16^. This work is quite inspiring not only because it confirmed the existence of STML and STDSs in MMF lasers, but also it fueled the study of ultrafast dynamics in dissipative nonlinear multimode systems^53^.

Subsequent to this milestone, studies on STML have flourished. The advancements include an improved understanding of the STML mechanism^18,54^, observations of spatiotemporal self-similaritons^17^, STDS molecules^55,56^, beam self-cleaning^57–59^ and mode-locking buildup dynamics^60–62^ in MMF lasers, and the realization of all-fiber STML lasers^19,63^ and mode field engineering in STML MMF lasers^64^. However, our understanding and control of STDS and STML still significantly fall behind the counterparts in SMF lasers. For example, the large mode area and content should allow the generation of ultrahigh pulse energy in fiber lasers, but the reported STML pulse energy is still lower than large mode area SMF lasers^16^^,65,66^ as a reference here. The active mode engineering in MMF lasers to achieve arbitrary mode profiles has not been achieved yet. Optical measurement techniques for spatiotemporal characterization are not very mature and user-friendly, which is also dragging our understanding of STDSs in MMF lasers. With these challenges tackled, MMF lasers can be used in many applications including distance metrology^67^, laser processing^68^, nonlinear spectroscopy^69^, optical tweezing^70^, and imaging in scattering media^71,72^.

Therefore, MMF lasers are a frontier for ultrafast optics with both challenges and prospects. More broadly, MMF laser is also a versatile platform to study nonlinear multimode photonics so as to strengthen our ability to manipulate photon behavior in multimode systems with nonlinearity complexity. And there are several review articles on nonlinear multimode photonics^53,73–76^; here we focus on STML in MMF lasers. We hope the review can serve as a general introduction of STML progresses and outline some interesting problems to work on in the near future. The review is organized as following. We first introduce the general theory and numerical modeling method of STDS propagation in MMF lasers in Section “Principle of STML”. We also categorized three STML regimes depending on the net intermode dispersion in this section. Section “Nonlinear spatiotemporaldissipative soliton dynamics“ covers the progress of STML including the spatiotemporal measurement techniques, nonlinear dynamics, mode field engineering and possible pathways towards higher pulse energy. Section “Discussions” is devoted to the outlook and conclusion of the review.

## Principle of STML

STML MMF lasers are high-dimensional systems. Many complex dynamics can exist in the laser and it is important to draw useful conclusions from rich observations to establish the basic principles for STML and STDSs. We introduce the general principles of STML including its modeling method, mode-locking condition and operation regimes in this section.

### Numerical modeling of STML

The STML dynamics in MMF lasers can be modeled by distributed models^77^ or lumped models^16^. The (3 + 1) dimensional complex cubic-quintic Ginzburg-Landau equation ((3 + 1)D CGLE) is an example of the distributed model^77^, where the motion equation of the normalized field envelope *ψ*(*x*, *y*, *z*, *t*) is written as,

1 $$\begin{array}{l}i{\psi }_{z}+{\displaystyle\frac{1}{2}}D{\psi }_{tt}+{\displaystyle\frac{1}{2}}({\psi }_{xx}+{\psi }_{yy})-\left({x}^{2}+{y}^{2}\right)\psi +\nu | \psi {| }^{2}\psi \\\quad\;\;\, +\,\gamma | \psi {| }^{4}\psi =i\delta \psi +i\varepsilon | \psi {| }^{2}\psi +i\beta {\psi }_{tt}+i\mu | \psi {| }^{4}\psi \end{array}$$

where the conservative and dissipative terms are listed in the left-hand-side and the right-hand-side of the equation, respectively, and subscripts of *ψ* represent derivatives. *x*, *y* are the transverse coordinates, *z* is the propagation direction and *t* is the fast time. *D* represents the dispersion (positive for anomalous dispersion) and the (*x*^2^ + *y*^2^)*ψ* term stands for the refractive index distribution of a graded-index (GRIN) MMF (step-index (STIN) MMF should have a different from); *ν* and *γ* are coefficients for the cubic and quintic nonlinearities, respectively. *δ*, *ε*, *β* and *μ* are the coefficients for linear loss (if negative), nonlinear gain (if positive), spectral filtering and saturation of the nonlinear gain (if negative), respectively. *ν*, *γ*, *ε* and *μ* are space-related terms. Eq. (1) allows solutions for both stable and pulsating STDSs and can be used to analyze the operation regimes of MMF lasers^77^.

Equation (1) averages intracavity dynamics within one round trip. In order to study STDS propagation in one round trip, a lumped mode can be used. In this model, STDSs are decomposed into a series of spatial modes and pass discrete cavity components (MMFs, saturable absorber (SA), spectral filter and spatial filter) in sequence, see Fig. 1b. The heart of the model is the generalized multi-mode nonlinear Schrödinger equation (GMMNLSE)^41,73,78,79^, which was first derived by Poletti and Horak^79^. In many cases, effects such as self-steepening, Raman scattering and higher-order dispersion can be neglected, while gain/loss must be considered for MMF lasers. Thus, nonlinear propagation in the MMFs of an STML laser can be modeled by an array of GMMNLSEs written as^73,80^,

2 $$\begin{array}{l}\frac{\partial {A}_{p}(z,t)}{\partial z}\,=\,i\left({\beta }_{0}^{(p)}-{\beta }_{0}^{(0)}\right){A}_{p}-\left({\beta }_{1}^{(p)}-{\beta }_{1}^{(0)}\right)\frac{\partial {A}_{p}}{\partial t}\\\qquad\qquad\; -i\frac{{\beta }_{2}^{(p)}}{2}\frac{{\partial }^{2}{A}_{p}}{\partial {t}^{2}}+\frac{g(z)}{2}{A}_{p}+i\frac{{n}_{2}{\omega }_{0}}{c}\mathop{\sum}\limits_{l,m,n}{S}_{plmn}^{K}{A}_{l}{A}_{m}{A}_{n}^{* }\end{array}$$

where *A*_*p*_(*z*, *t*) is the electric field envelope for mode-*p* (mode-1 is the fundamental mode and *l*, *m*, *n* stand for mode number too), ${\beta }_{r}^{(p)}(r=0,1,2)$ is the *r*th order Taylor expansion of the propagation constant for mode-*p*; *g*(*z*) is the saturated gain for the active MMF and is zero for the passive MMFs. To take spectral and spatial dependence of gain into account, signal amplification is computed in the frequency domain as, ∂_*z*_*A*(*x*, *y*, *z*, *ω*) = *g*_0_(*ω*)*A*_*p*_(*x*, *y*, *z*, *ω*)/[2(1 + ∫∣*A*(*x*, *y*, *z*, *t*)∣^2^*dt*/*I*_sat_)], where *A*(*x*, *y*, *z*, *t*) = ∑_*p*_*F*_*p*_(*x*, *y*)*A*_*p*_(*z*, *t*) is the total field of the STDS (not mode-resolved pulse), *F*_*p*_(*x*, *y*) is the transverse mode profile of mode-*p* and *A*(*x*, *y*, *z*, *ω*) is the spectrum of the STDS; *g*_0_(*ω*) and *I*_sat_ are the small signal gain spectrum and the saturation intensity for the active MMF, respectively^16,18^. The last term represents the Kerr nonlinearity with *n*_2_ being the nonlinear refractive index (*n*_2_ = 2.3 × 10^−20^ m^2^/W for silica) and ${S}_{plmn}^{K}$ being the inverse of the effective mode area with mode overlap accounted^73^. The Kerr nonlinearity can bind modal pulses and enable energy exchange among them. To generate 3D ultrashort pulses, a space-dependent and the power-dependent (i.e., SA) is included^16,18^. Note that SA will change the pulse shape, thus shifting the pulse center position^81^.

Spatial filtering (or spatial-mode-dependent loss) can also align modal pulse positions for self-consistent propagation^16,73^. It can be implemented by a pinhole, spatial coupling from free space into fibers or fiber splicing^16,18,23^. The latter works by offset splicing between identical fibers or splicing between fibers with different core diameters. Such a splicing can not only excite high-order modes but also constitute a spatial filter. This is because many STML lasers used single-mode (or quasi single-mode) gain fibers with a core diameter about 10 μm and passive MMF with a large core diameter (e.g., OM4 or OM1 fibers have core diameters of 50 μm or 62.5 μm, respectively)^16,17,22,55,57,81,82,83^, and splicing between them causes spatial filtering. Note that the small-mode-area fiber may limit the gain and pulse energy scaling for STML lasers. An *n* × *n* matrix *M* can be used to model the pinhole, fiber splicing or spatial coupling based spatial filters as *A*_out_ = *MA*_in_, where *A*_in_(*A*_out_) is a *n* × 1 vector representing the mode-resolved field before (after) coupling. The diagonal elements stand for the intramode loss and the off-diagonal elements of *M* stand for energy exchange between different modes. Propagation in one round trip completes after passing all the components, and round trip iteration continues till the convergence of an STDS solution.

Both models can be used to analyze STML dynamics, reaching reasonable agreement with measurements; however, the computation complexity is usually high. And quantitative agreement with measurements remains a challenge, considering the difficulty in modeling the high dimensional laser parameters accurately enough.

### STDS in MMF lasers

Earlier studies on pulse propagation in single pass MMFs have established the existence of the multimode solitons^36–39^. Such multimode solitons are formed when the nonlinearity balances the chromatic dispersion as well as the intermode group velocity dispersion^36–38^ (see also Eq. (2)). They differ from solitons in SMFs as the ultrashort pulses within MMFs are made up of several modal pulses that can have different spectra. STDSs in MMF lasers are also different from light bullets in free space nonlinear systems^84–87^, as mode confinement of the MMFs gives limited and discrete spatial modes (i.e., diffraction is naturally counteracted).

**Fig. 2:**
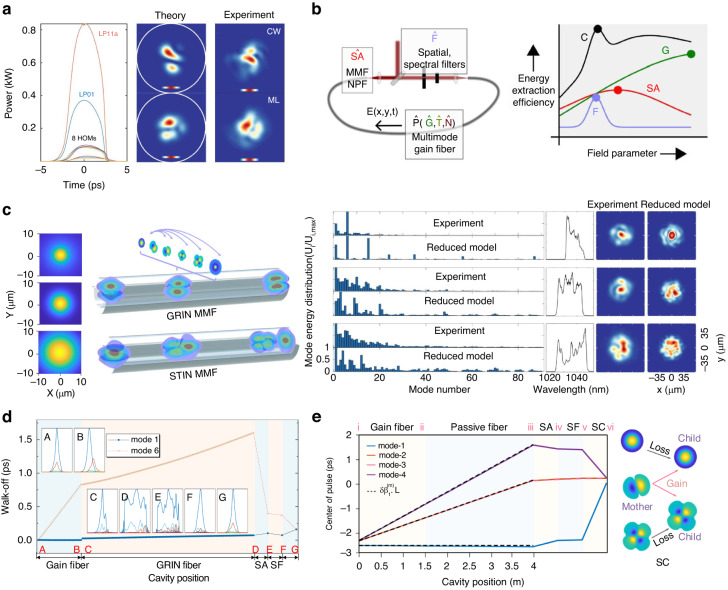
STDS generation in MMF lasers. **a** Simulated modal pulse and comparison of the spatial beam profile between theory and experiment^16^. Adapted with permission from ref. ^16^ © American Association for the Advancement of Science. **b** Top left panel: Illustration of the attractor dissection theory. Different physical effects are simplified as projection operators. SA, F, and P are operators standing for spatiotemporal saturable absorber, intracavity spatial filter and gain dynamics. Top right panel: steady STDS is formed by the overall effects of the operators, and may operate in a spatial filter dominated state. Bottom panel: experimentally measured mode content and beam profile and their comparison with the attractor dissection model^54^. Scale bar: 35 μm. Adapted with permission from ref. ^54^ © 2020 Nature Publishing Group. **c** Comparison of spatiotemporal pulse propagation in GRIN and STIN MMF based fiber lasers. For GRIN MMFs, the walk-off for modal pulses is relatively small and the Kerr effect can be important to bind them. The large intermode dispersion of STIN MMFs cannot be balanced by the Kerr effect, and modal pulses walk off strongly. The inset shows a comparison of the fundamental mode between SMF, GRIN MMF and STIN MMF, see ref. ^23^. Adapted with permission from ref. ^23^ © The Optical Society. **d** Simulated STDS dynamics in a MMF laser with a large intermode dispersion gain fiber. Mode-1 and mode-6 walk off quickly in the STIN gain fiber and saturable absorber pulls back the walk-off^18^. Reproduced with permission from ref. ^18^ © American Physical Society. **e**, Intracavity change of the moal pulse center position in all STIN MMF laser^23^. Modal pulses walk off at a rate determined by $\delta {\beta }_{1}^{(m)}$ in the MMFs and spatial coupling contributes strongly to compensating the walk-off. The right panel is an illustration of a proposed mother-child coupling picture to understand STML in all STIN MMF lasers^23^. SA saturable absorber, SF spectral filter. Reprinted with permission from ref. ^23^ © The Optical Society

The first experimental demonstration of the STDS leveraged the careful designed laser dissipation to realize self-consistent propagation in both time and space domains^16^ (Fig. 2a). Akin to the all-normal-dispersion SMF lasers where a spectral filter is used to shorten the highly chirped pulses for self-consistent propagation in the time domain^88^, spatial filter is important to align the modal pulses.

The formation of STDS can be understood by the attractor dissection theory^54^. This theory is based on the maximum gain (or minimum loss) principle for lasers^89^ that means an STDS tends to adjust itself to receive higher gain and lower loss. In the attractor dissection theory, different effects and cavity components are simplified as an operator. For example, the nonlinearity can be simplified as an operator to represent the overall Kerr nonlinearity on the intracavity field in one round trip, see Fig. 2b. The other effects including spatiotemporal gain, dispersion, spatiotemporal SA, and spatial and spectral filtering can also be represented by corresponding projection operators. The STDS corresponds to a solution which attains the maximum gain (minimum loss) after many round trip iterations for these operation projections. The path of a laser to reach a steady state can be curved, but the spatiotemporal attractor is usually fixed for a given system. Different operators may have different contribution to the final attracted solution. For example, the MMF laser can be dominated by the spatial filtering effect when having a strong spatial filtering for STML (see the top right panel of Fig. 2b). And the MMF laser may transition from the spatial filter dominated state into a saturable absorber dominated state by adjusting the pinhole size^54^.

Through the attractor dissection theory, the complex lasing dynamics in a MMF laser can be simplified as an optimization problem to find the highest gain solution. Since the attractor dissection theory only considers the overall effects in the cavity instead of concrete effects by solving the computationally complex GMMNLSE, it reduces the computation complexity greatly. The mode content calculated by this model is in reasonable agreement with the measurements, see the bottom panel of Fig. 2b. Therefore, it can be an efficient theory to understand STML in MMF lasers and paves a way for convenient MMF laser design. However, it is still worth studying concrete roles of different laser cavity components to understand their impact on the STML dynamics in detail. For example, how space-dependent gain saturation and saturable absorption influence the STDS properties. From this perspective, the lumped model introduced in Section. “Numerical modeling of STML” still has its own value.

An interesting question for STDS is how the intermode dispersion is balanced. Different spatial modes propagate with different phase and group velocities in the MMFs. For multimode solitons in a GRIN fiber, weak group velocity difference can be largely balanced by the Kerr effect^38,78^. For the STDS in GRIN MMFs dominated lasers, the walk-off for mode-resolved pulses (or modal pulses) is relatively small and the Kerr effect can be important to bind them, see body diagrams in Fig. 1b and temporal-domain interpretation in Fig. 1c; and spatial filter also contributes to counteracting weak walk-off in GRIN MMF lasers^16,54^. When the modal walk-off is large (e.g., in STIN MMF lasers), the Kerr effect cannot bind the modal pulses together and they can walk off strongly. Since walk-off is not balanced by the Kerr nonlinearity, dissipation including SA, spectral and spatial filters should be responsible for compensating the large walk-off (which will be discussed in detail in the next subsection).

### STDS under large intermode dispersion

Rare-earth doped GRIN fiber is rare, whereas STIN gain fiber is more commercially mature. Hence, it is a natural question whether STML can be realized in MMF lasers comprising STIN MMFs, which have much larger intermode dispersion than GRIN MMFs. If it is possible, the operation regime of STML can be extended considering the distinct dispersion and nonlinear properties of STIN MMFs, thus richer STDS dynamics can be envisioned. Moreover, the mode area of a STIN MMF tends to be larger than a GRIN MMF, which will lead to higher pulse-energy (Fig. 2c)^73^. Therefore, there is a strong motivation to investigate STML in MMF lasers comprising STIN MMFs that have larger mode area and intermode dispersion.

Along this line, we demonstrated STML with large intermode dispersion in a MMF laser including a long active STIN MMF^18^. The STIN MMF introduces large walk-off between spatial modes (for example, ~1 ps/m intermode dispersion between LP01 mode and LP21 mode, versus 0.1 ps/m for typical GRIN MMFs) in the laser. STML can still be observed in the MMF lasers with an STIN gain fiber length up to 0.6 m. The output mode profile can be controlled and a near-LP11-mode output was observed. Besides initializing STML, SA was also found to be important to counteract the large intermode dispersion. Simulations show the relative modal pulse positions between LP01 mode and LP21 mode changed quickly in the STIN gain fiber (Fig. 2d). This walk-off further increased to about 1.5 ps in the passive GRIN MMF. Then, the SA pulls the walk-off back to below 0.5 ps. Finally, the spatial filter resets the pulse position and ensures self-consistent propagation. The role of SA that counteracts walk-off can be understood as following. Taking LP01 mode and LP21 mode for example, before the pulse passing the SA, two modes have different center positions. Space-dependent SA leads to different nonlinear loss for modal pulses and a redistribution of modal energy, shifting the relative position between them (middle panel in Fig. 1c). It should be noted that the walk-off compensation capability of a SA is limited to the pulse width range and worked well for the laser reported in ref. ^18^. For cavities with larger intermode dispersion, the role of SA is less significant and spatial coupling/filtering plays a more critical role.

By replacing the remaining passive GRIN MMFs with passive STIN MMFs, we have further demonstrated STML in an all STIN MMF laser^23^. The net intermode dispersion of the all STIN MMF laser (evaluated between the LP01 and the LP11 mode at 1060 nm) is about three times of the above hybrid STIN and GRIN MMF laser^18^. Experimental and numerical results show that pulses belonging to different modes walk off significantly in the all STIN MMF laser and it is the spatial filtering (implemented by spatial coupling from free space into a MMF; see ‘Spatial coupling’ case in Fig. 1b for illustration) that compensates the large walk-off (Fig. 2e). In short, the all STIN MMF laser can allow a single mother-mode, while the seed for other child-modes for the next round trip comes from the mother-mode (see right panel in Figs. 1c and 2e). Thus, self-consistent propagation can be guaranteed despite the large intermode dispersion. In principle, this mother-child-coupling scheme can enable pulse generation with a single repetition rate in MMF lasers with infinitely large intermode dispersion under an appropriate spatial coupling condition.

## Nonlinear spatiotemporal dissipative soliton dynamics

Multimode nonlinear systems allow richer nonlinear interactions than single-mode systems. The additional space dimension inspires new physical pictures and theoretical tools to understand nonlinear dynamics in ultrafast lasers. These nonlinear dynamics can be relatively complicated but is essential to understand ultrashort pulse propagation in high-dimension nonlinear systems and to control STDSs in MMF lasers.

### Measurement of spatiotemporal dynamics

For SMF lasers, usually time domain and frequency domain measurements are needed to characterize the mode-locking state. For MMF lasers, the space dimension constitutes a new dimension that needs to be characterized. The early techniques to characterize the STML are spectral filtering and spatial sampling^18,55,73^. As a signature of STML, the mode profile will change when selecting different portions of the spectrum by spectral filtering, while the spectrum changes when spatially sampling different part of the output beam. These measurements are usually used to validate that the STML state occupies multiple spatial modes^16,55,61,81^. Nevertheless, they only provide rough estimation of the mode content. More rigorous mode decomposition is needed to understand the mode content quantitatively^54,90–99^.

**Fig. 3:**
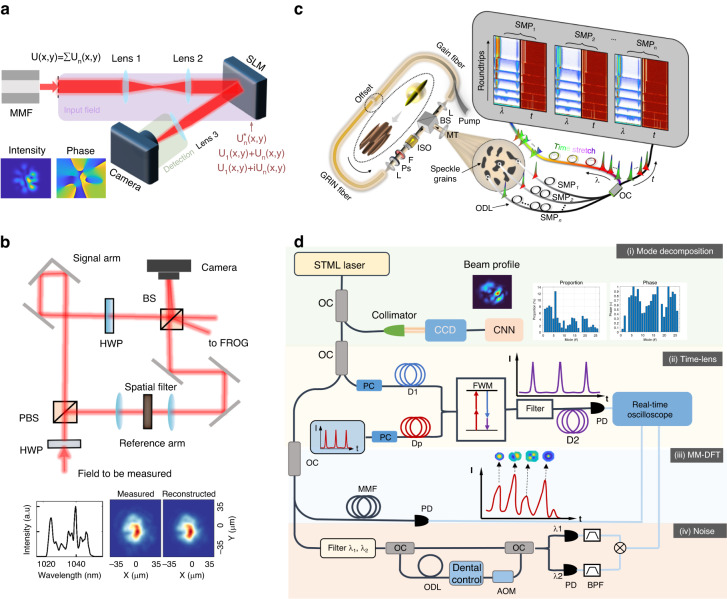
Characterization methods for spatiotemporal dissipative soliton (STDS). **a** Mode decomposition of a multimode output beam *U*(*x*, *y*) by the correlation filter method (CFM)^90^. Eigenmodes of the MMF (${U}_{n}^{* }(x,y),{U}_{1}(x,y)+{U}_{n}(x,y),{U}_{1}(x,y)+i{U}_{n}(x,y)$) are encoded a spatial light modulator (SLM) in sequence. The SLM output is then Fourier transformed by a lens and detected by a camera or single pixel detector for modal control retrieval. Adapted with permission from ref. ^90^ © The Optical Society. **b** The interferometric digital off-axis holography setup for mode decomposition experimental result is shown at the bottom^54^. Complex field of the input pulse at different signal arm delays are measured to decompose the STDS. Adapted with permission from ref. ^54^ Copyright 2020 Nature Publishing Group. **c** Schematic diagram of the real-time multispeckle spectral-temporal (MUST) measurement system for characterization of STDSs^62^. Spatiotemporal signals from multiple speckle grains are collected by different single-mode fiber probes, which are then temporally multiplexed by optical delay lines. The multiplexed signal is split into two branches, one of which is directly detected, while the other branch is launched to a dispersive Fourier transform (DFT) unit for real-time measurement. Reprinted with permission from ref. ^62^ © Springer Nature. **d** A proposed system to characterize STDSs. It includes (i) a machine learning based subsystem for mode decomposition, (ii) a time lens subsystem for real-time time domain observation, (iii) a multimode dispersive Fourier transform (MM-DFT) subsystem for real-time spectrum observation, and (iv) a delayed self-heterodyne subsystem for timing jitter characterization^122^. OC optical coupler, CCD charge-coupled device, CNN convolutional neural networks, PC polarization controller, FWM four-wave-mixing, PD photodetector, AOM acoustic optical modulator, ODL optical delay line, BPF bandpass filter

There have been several techniques to deconstruct the multimode optical field and retrieve the mode content^90–98^. One example is the spatially and spectrally (*S*^2^) resolved imaging technique, which utilizes propagation in a MMF and intermode dispersion to separate different spatial modes for mode retrieval^91^. Another technique is the correlation filter method (CFM), see Fig. 3a. To decompose mode-*n* *U*_*n*_(*x*, *y*), the complex fields ${U}_{n}^{* }(x,y),{U}_{1}(x,y)+{U}_{n}(x,y),{U}_{1}(x,y)+i{U}_{n}(x,y)$ are encoded on a spatial light modulator (SLM) in a sequence^92,93,100^. The corresponding outputs are recorded by a camera and processed to retrieve the power ratio and relative phase of mode-*n* with respect to mode-1. Other techniques involve measuring the complex amplitude of the multimode field using methods like delay-scanning off-axis digital holography^54,94,95,99^ (Fig. 3b), as well as the so-called TERMITES^101^, SEA TADPOLE^102^ techniques and other variants^103^. These methods has been successfully used to characterize STDSs from MMF lasers. In particular, delay-scanning off-axis digital holography based mode decomposition has been used to retrieve the mode content and verify the attractor dissection theory^54^.

However, these methods are relatively complicated. A relatively simple way to decompose modes is to measure the output mode intensity only and decompose the mode by machine learning^96,96–98^. Currently, it can only be used to decompose a relatively small mode number (less than 10 modes)^96,96–98^. More advanced algorithms are needed to implement the mode decomposition for larger mode number cases. All the mentioned methods need a relatively long processing time and not suitable for real-time observation, which is critically important to understand STML dynamics.

3D lightwave dynamics have been measured in real-time by using single-shot imaging techniques such as STRIPED FISH^104^, STS-CUP^105^, and CUST^106^. However, these techniques have a limited number of continuous frames, which hardly measure STDS dynamics over a long time scale. Recently, a beam mapping technique reaching picosecond time resolution was demonstrated to study intrapulse beam self-cleaning dynamics^107^. However, the mapping relied upon mechanical scanning and is not suitable for real-time observation of non-repetitive events. Speckle-resolved measurements of the multi-mode output can be a relatively simple space-resolved approach to measure non-repetitive STDS dynamics in real-time over a large number of round trips^62^. The example shown in Fig. 3c used speckle-resolved sampling and dispersive Fourier transform (DFT) technique^108^ for real-time observation of spectral dynamics for STDSs^60,62,109^.

However, the SMF-based DFT measurement does not reflect the complete modal components, as the spatial sampling is relatively coarse. Mode-resolved real-time observation of multimode optical fields is still challenging. Here we propose a MMF-based DFT technique for mode-resolved real-time observation of STDS. Mode content can be retained when coupling into the fiber by selecting an appropriate MMF. Since the intermode dispersion is much larger than the chromatic dispersion, MMFs with large intermode dispersion (for example, STIN MMF) can separate spatial modal pulses via intermode dispersion. The output spectrum can be relatively complicated when the MMF is long. Fortunately, neural networks can be used to reconstruct the spectrum^110^.

In addition to DFT-based real-time spectrum measurement, real-time measurement of the temporal waveforms is also important^9,111^. Temporal lens, which stretches an ultrashort pulse in the time domain^112,113^, can enable single-shot measurement of the waveform with femtosecond-level resolution^9,111,114,115^. This measurement technique can also be used to measure fast-varying temporal dynamics of STDSs in MMF lasers. For example, STDSs under large intermode dispersion and multimode bound states are often not single-peaked, and time lens may be used to measure the formation of these temporal structures.

Another key property of STML lasers that remains to be characterized is the noise performance. High periodicity of the pulse stream (i.e., low timing jitter) is a prerequisite for precise timing and optical frequency comb applications. The ultralow timing jitter of SMF lasers has been used in many applications including optical frequency division for ultralow noise microwave synthesis^116,117^, timing synchronization and dissemination^118^ and distance metrology^119,120^. Whether STML lasers can achieve similar stability remains an open question. Careful measurement of timing jitter in STML using phase noise analyzers as well as the heterodyne^121,122^ and balanced optical cross-correlator techniques^118,123^ can reveal the noise property of MMF lasers and pave the way to reduce timing jitter for STDSs. Theoretical work on the noise dynamics is also needed to better understand STML in MMF lasers. By combining the above measurement techniques, we propose a characterization system shown in Fig. 3d, which can enable real-time measurement in the frequency and time domains, as well as static mode decomposition in the space domain and timing jitter analysis.

### Spatiotemporal dynamics of STDSs

**Fig. 4:**
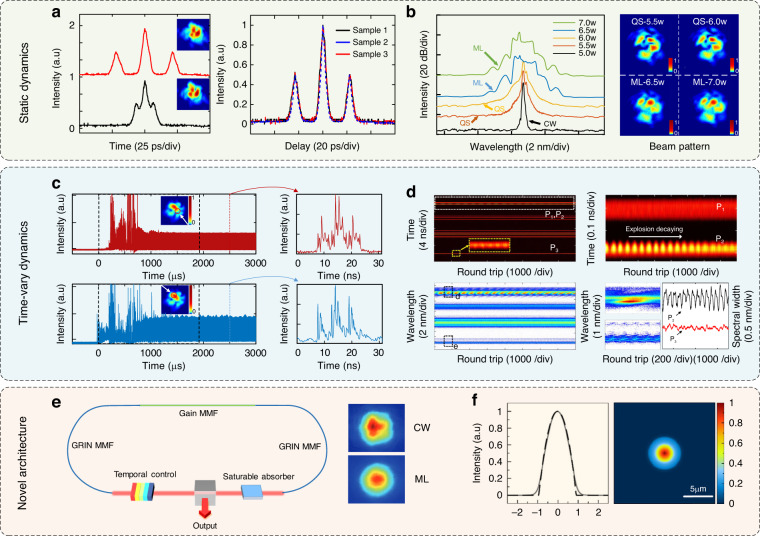
Spatiotemporal dissipative soliton dynamics in MMF lasers. **a** Left: observation of spatiotemporally bounded states with different separations and corresponding beam profiles. Right: autocorrelations of pulses sampled at different beam positions^55^. Adapted with permission from ref. ^55^ © The Optical Society. **b** Transition between Q-switching (QS) and STML by varying pump power. Spectra of the laser output with increasing pump power, covering the continuous wave (CW), QS, and STML regimes^61^. ML mode-locking. Reprinted with permission from ref. ^61^ © The Optical Society. **c** Buildup dynamics of a multi-pulses STDS state probed at two spatial sampling points, which shows distinct buildup routes. The figures in the right panel shows the DFT time-stretched pulses, indicating different spectrum features for two different channels^60^. Adapted with permission from ref. ^60^ © The Optical Society. **d** Observation of STDS explosion in a MMF laser. In the multipulse cluster, in addition to the ordinary solitons (P1), a spectrally and temporally explosive pulse (P2) is observed^62^. Adapted with permission from ref. ^62^ @ Springer Nature. **e** Dispersion-managed cavity enabled beam self-cleaning in STML fiber lasers. Right panel: measured output beam profile for CW and STML states^57^. Adapted with permission from ref. ^57^ © 2020 SPIE. **f** Self-similar STDS in an STML fiber laser. Simulated pulse shape (solid) can be fitted by a parabolic pulse shape (dashed)^17^; the output beam is a quasi-fundamental-mode. Reprinted with permission from ref. ^17^ © The Optical Society

Using the measurement techniques mentioned above, spatiotemporal dynamics of STDSs can be characterized. Different from conventional solitons (sech-shape), dispersion-managed solitons (Gaussian-shape) and similaritons (parabolic-shape) in SMF lasers, STDS may have structured shapes^18,22,99^. Unlike SMF lasers, nonlinear intermode interaction leads to energy transfer among spatial modes. Therefore, STDS can have distinct dynamics from the counterpart in SMF lasers. In addition to single-pulse states, multi-pulse states, including harmonic mode-locking and bound states (or STDS molecules)^55,56,81^ have been observed in MMF lasers. The multi-STDS spatiotemporal characteristics (e.g., pulse separation in a bound state) can be controlled by varying the lasing condition^55,56,109,124^, see Fig. 4a. Spatially sampled autocorrelation measurements showed that temporal separations of the bound state are the same for pulses spatially sampled at different positions of the output beam, suggesting all the modal pulses are bounded with a uniform pulse separation^55^ (Fig. 4a).

In SMF lasers, time-varying dissipative soliton dynamics widely exists and helps understand temporal dissipative solitons. For example, mode-locking buildup dynamics^125,126^, pulsating (breathing) dissipative solitons^127,128^, soliton explosion^129,130^ and rogue waves^131,132^ all add to our understanding of single-mode lasers. Single-shot measurements of these time-varying behaviors have become possible with the use of the DFT and time lens techniques in SMF lasers. Similarly, by employing spatial sampling and the DFT technique, space-resolved STDS dynamics were also measured^60,62^. During the STML building up, different modes can evolve differently, i.e., reaching the attractor via different routes. Specifically, the evolution patterns during the relaxation oscillation as well as Q-switching states differ from mode to mode. In addition, multiple pulses with different energies can exist in a MMF laser; furthermore, the pulse energy ratio between them can vary for different spatially sampled positions^60^ (Fig. 4b). Depending on the pump power, reversible Q-switching and STML transition was also observed^61^. A critical bistability between the multimode Q-switching and STML can also be realized under appropriate cavity spatial coupling and nonlinear polarization rotation states (Fig. 4c). As a possible route towards spatiotemporal chaos, period-doubling bifurcation in STML has also been reported in MMF lasers^133^. A simple iterative model considering the mode-dependent saturable absorption effect is proposed to understand the role of spatiotemporal SA for the unique spatiotemporal characteristics of period-doubling bifurcation in the MMF laser. Space-dependent STDS explosion has also been observed^62^, giving new opportunities to understand strange dissipative soliton attractors^6^ (Fig. 4d).

### Mode field engineering

Mode field engineering and wavefront shaping are important to beam self-cleaning^33^, controlling nonlinear wave interactions^49,134^ in MMFs. STDSs with engineered mode field may enable many applications including optical storage^135^, super-resolution imaging^136^, 3D laser lithography^137^. These applications can have quite different requirement on the mode profile. Nevertheless, customized mode engineering for STML lasers has not been mature yet.

Since high beam quality is usually needed for applications requiring tightly focused beams (e.g., femtosecond laser processing^68^, nonlinear spectroscopy^69^), attaining quasi-fundamental mode in MMF lasers has been a longstanding goal for STML. Beam self-cleaning, that is first observed in single-pass MMFs^31^, is a promising method to achieve this goal. Several models have been proposed to understand the observed self-cleaning^28,31,58,138–140^. Recently, Rayleigh-Jeans distribution, an analogy to a thermal equilibrium in thermodynamics, was proposed to understand the self-cleaning in GRIN MMFs^140^. Self-cleaning and a near single-mode output beam have also been reported in STML MMF lasers^57^, see Fig. 4e. By well designing bandpass filter and fiber length, a low chirp parabolic pulse with near-Gaussian beam quality (M^2^ ≤ 1.4) was observed in a MMF laser^17^. This work verifies the possibility to obtain high-power, high-quality beam profile ultrashort pulses from MMF lasers. In addition, Ytterbium absorption, leading to enhanced lose for higher-order modes, could be a possible reason for self-cleaning in active fibers^58^. It is worth mentioning that combining the gain rate equations with GMMNLSE may contribute to revealing the underlying mechanism.

Another way to control the mode field agilely is to insert an SLM in the laser cavity. For example, an intracavity SLM was used to produce almost single-mode output using the genetic algorithm (but in a continuous wave state)^64^. Nevertheless, the generation of arbitrary output beam profiles has not been demonstrated yet. Typically, an SLM only encodes the phase of the input. Adding a grating pattern to the phase coding can be used to produce effective amplitude modulation^91,141,142^. However, this method only works for narrow bandwidth cases, because broadband pulses will experience dispersion induced by the grating pattern, complicating the actual modulation by the SLM. Therefore, it can be challenging to encode both complex phase and amplitude modulation using a phase-only SLM for STML pulses. Moreover, the damage threshold of SLM presents another practical issue. Digital micromirror devices (DMDs) or diffraction optical elements may be used to increase the damage threshold. And a combination of SLM and DMD may enable simultaneous phase and amplitude encoding to control STML in a programmable way. Since spatial control is essential for spatiotemporal focus in scattering media (complex mode will be needed)^71,72^, such STDSs with programmed beam profile may be used for nonlinear microscopy in scattering samples.

In addition, other optical components like metasurfaces^143,144^, q-wave plates^145^ or mode converters^146,147^ have been integrated into SMF lasers to produce exotic lasing modes. It will be interesting to investigate how these devices can modify the STML dynamics in MMF lasers, which brings new opportunities for transverse mode control and mode engineering^148^.

### Towards higher energy

Modulating a single-mode field outside laser cavities can also produce a mode-engineered field. However, STML offers a potential route towards high pulse energy with engineered beam profile. Nonlinearity is a double-edged sword for pulse energy scaling in fiber lasers. Various nonlinear pulse shaping mechanisms have been developed for SMF lasers^149^. Conventional soliton fiber lasers typically have pulse energy less than 1 nJ^150–152^; dispersion-managed soliton can be used to increase the pulse energy to above 10 nJ^153–156^; self-similar pulse generated in the normal dispersion regime can also generate 10-nJ-level pulses^157–159^; dissipative soliton in the all-normal-dispersion fiber lasers can further increase the energy to several tens of nJ^88,160–162^. Large-mode-area photonic crystal fibers (PCFs) with a low nonlinearity can be used to increase the pulse energy to hundreds of nJ^163,164^ (but PCFs needs careful maintenance). Recently, PCF-based Mamyshev oscillators have brought the pulse energy in SMF lasers to the μJ regime^66^.

SMF laser experiments show the accumulated nonlinear phase shift within a laser cavity is usually limited to ~10*π*^165^. Given the loose mode confinement and the relatively large mode area of MMFs, which is more robust than large-mode-area PCFs, the single pulse energy in STML can be as high as ~150 nJ^16,166^. By further increasing mode area of gain fibers and lowering intracavity loss, higher pulse energy could be possible. In addition, the nonlinear pulse shaping toolbox discussed above for SMF lasers has not been fully used for MMF lasers yet. Exciting opportunities should arise by releasing these potentials and μJ-level pulse energy may be available from MMF lasers without external amplification.

Among these possibilities, a promising approach is to bridge Mamyshev oscillators and MMF lasers. It can take advantage of the large mode content and low nonlinearity of MMFs to further enhance the output pulse energy from Mamyshev oscillators. Initial attempt to combine them has led to STML based on Mamyshev regenerators^22,83^; however, the demonstrated pulse energy is less than 20 nJ in these reports. Higher power may be possible by lowering the cavity loss, e.g., using all-fiber multimode Mamyshev oscillators or using gain fiber with a larger mode volume. Further work is needed to achieve higher pulse energy in MMF Mamyshev oscillators.

As mentioned above, all-fiber designs offer a viable solution to lower losses and increase the output power. Indeed, nonlinear multimode interference (NL-MMI) filter using a SMF-MMF-SMF structure^167–169^ can be used as a spectral and spatial filter for STML in MMF lasers^19,63,82,170,171^. By precisely tuning the NL-MMI filter, tunable dual-wavelength STML can be realized with two wavelength emissions showing distinct characteristics^170^. Nearly 500 mW power output with optimally balanced two-color intensities was realized using the NL-MMI for mode-locking in a MMF laser^172^, which provide a promising synchronous multi-color pulse source for THz-wave generation and Raman scattering spectroscopy^173,174^. The damage threshold for all-fiber devices needs to be considered for high power applications, but it is promising to realize high power MMF lasers with a compact form factor and high robustness.

Besides beam quality, pulse quality is also important for applications. The envelope of STDSs in MMF lasers may be structured^16,18^. Similaritons with a parabolic shape and a linear chirp based on pulse propagation in the normal dispersion regime is a feasible approach to obtain high pulse quality^175^. Such a type of pulse has also been observed in an STML MMF laser with a narrow spectral filter^17^ (Fig. 4f). Such a combination of good beam quality and pulse quality is compelling and deserves further exploration. Generating energetic STDSs with high pulse quality direct from MMF laser could eliminate the power amplification system and higher intracavity pulse energy can reduce the noise of the pulse stream^176^. As an aside, continuous wave (CW) emission is observed to coexist with STDSs in MMF lasers^16,18^, which can be a limiting factor for pulse energy scaling. By increasing the modulation depth of the SA, CW emission can be eliminated^83^.

## Discussions

**Fig. 5:**
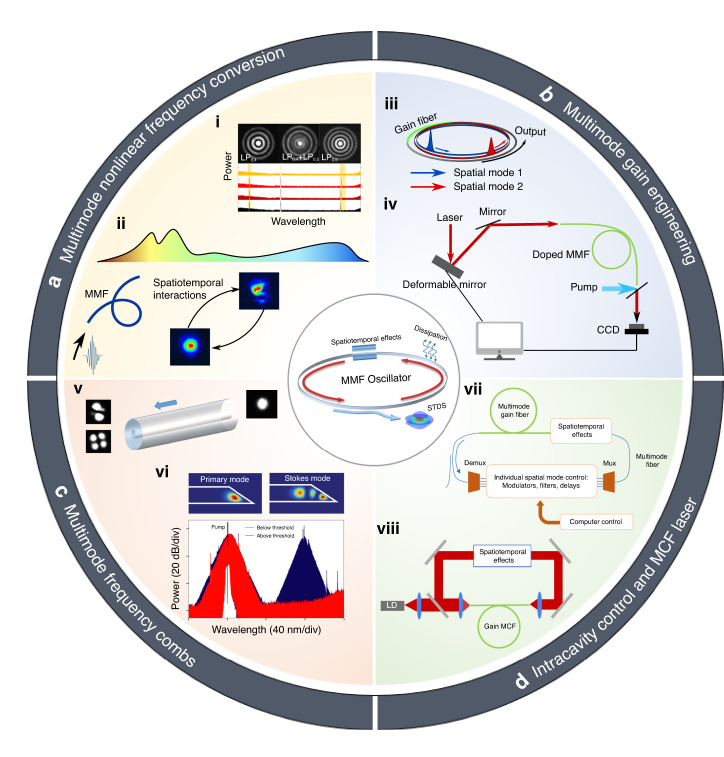
Prospects of spatiotemporal mode-locking (STML) lasers. **a** Multimode nonlinear frequency conversion in STML lasers, including i intermode four-wave-mixing (FWM)^179^ and ii supercontinuum generation^26,27,42,44^. i is adapted with permission from ref. ^179^ © The Optical Society. **b**, Multimode gain engineering, which may enable iii spatial-mode-multiplexed multi-comb generation in a single MMF laser. iv Moreover, shaping the light field in the gain MMF may optimize the gain extraction from the media for STDSs^134^. **c** Observation of STML in v a high-Q Fabry-Pérot (FP) microresonator made of few-mode graded-index MMF^20^. vi Stokes soliton reported in a high-Q silica microcavity, with the primary pulse and the Stokes pulse hosted in different spatial modes^183^. vi is adapted with permission from ref. ^183^ © 2017 Nature Publishing Group. **d**, vii Full intracavity control of spatial modes by mode multiplexing (Mux) and demultiplexing (Demux) proposed in ref. ^149^. viii Multi-core fiber (MCF) mode-locked lasers, where the core-coupling in the transverse coordinate can also be important to the mode-locking dynamics

The presence of the spatial dimension offers great potential to control STDSs. For example, STML lasers emitting in the telecom, visible and mid-infrared bands besides the commonly used 1 micron band can be invaluable for various applications^21,177,178^. In addition to using other rare-earth-doped MMFs, multi-color emission in STML MMF lasers may be possible by tailoring the intermode phase-matching conditions, as shown in a single-pass MMF (Fig. 5-i)^179^. Harnessing nonlinear intermode interaction and spectral broadening in MMFs^26,27,42,44^ to generate ultrabroadband output from STML lasers is another option to cover other emission bands (Fig. 5-ii). The spatial complexity can also be leveraged to generate mode-multiplexed frequency combs from a single MMF laser (Fig. 5-iii). Compared to multi-comb from a single SMF laser cavity^180^, such multi-comb states can have a larger repetition rate difference (up to 10 kHz may be possible by leveraging multi-mother-modes with a relatively large mode index difference). This large difference is favored for dual- or triple-comb applications, as it can increase the acquisition rates for spectroscopy and ranging^181,182^. Engineering the mode content of STDSs may enhance the effective gain in the active MMF and boost the pulse energy^134^ (Fig. 5-iv).

In parallel, STML in coherently pumped passive few-mode fiber cavities has been demonstrated^20^ (Fig. 5-v). Stokes solitons have also been observed in on-chip silica high-Q microresonators, where the primary and the Stokes pulses occupy different spatial modes^183^. However, these two pulses are centered at two quite different frequencies (Fig. 5-vi). STDSs sharing a same pump and similar center frequencies remain to be demonstrated in chip-scale microresonators. Different from MMF lasers with active gain, parametric gain in coherently pumped systems can lead to distinct dynamics. Comparing similarities and differences in the two systems may shed new insight into STML.

Mode multiplexing/demultiplexing devices have been widely used in optical communications^184,185^. Similar devices can also be used in MMF lasers to control spatial modes independently as proposed in ref. ^149^ (Fig. 5-vii). Such a full control is relatively complicated but is quite promising for agile STDS control, in addition to the potential SLM-based approach discussed in Section “Mode field engineering”. An intermode dispersion management STML cavity has been demonstrated using coupling long-period fiber grating^148^. Moreover, multicore fiber (MCF) lasers have emerged as a promising candidate for ultrahigh power solid-state lasers^186^. Mode-locking in them is possible but quite challenging^186^. Mode-locking dynamics may be impacted by optical coupling between neighboring cores, which differs from spatial mode interaction within one core. Coupling in the space coordinate for MCFs may be controlled by varying the core separations, which may be easier than the above mode-demultiplexing-based STML control. Hence, MCF lasers may be another platform to explore mode-locking dynamics impacted by the space coordinate (Fig. 5-viii).

Intracavity control and advanced measurement techniques (including data-driven and computationally intensive modeling and control techniques^187^) for multimode optical fields are both highly needed to tailor spatiotemporal pulses from MMF lasers. Their combination can also enable observation of exotic spatiotemporal dynamics (for example the formation of 3D pulses with both the beam profile and the pulse-shape preserved in MMF lasers). The high repetition rates of MMF lasers (thus increasing the single-shot data acquisition rate) and the spatial dimension makes MMF lasers an excellent platform to understand the nonlinear dynamics. These will ultimately add to the fundamentals of laser and light. The engineered output beam may be used to enable novel light-matter interaction experiments.

Such applications would require customized 3D pulses. The customization is a nontrivial and challenging inverse problem for STML lasers complicated by spatial-mode-coupling, nonlinearity, and dissipation in MMF lasers^188^. On the other hand, engineered beam is not always needed and spatiotemopral coherence can be used as a knob to enable new applications^189^. The concept of incoherent dissipative solitons has been established in SMF lasers^190^. With the added spatial dimension, incoherent STDS is also worth investigation. For example, revealing spatial mode interaction and modal energy exchange (resulting in beam profile variation) in soliton explosion and rogue wave states will add to our understanding of STDS. The time-varying beam of incoherent STDSs could be used for speckle-correlation-based imaging^191,192^ and wavefront shaping^193^. Low coherence for incoherent STDSs in time, frequency and space domains may also be used in chaotic Lidar^194^, optical cryptography^195^ and optical coherence tomography^196^ where low coherence turns out to be an advantage. Compared to single-mode incoherent dissipative solitons, the added spatial incoherence can offer new opportunities in these scenarios. Hence, spatial mode content and coherence can be engineered towards different objectives. Full spatiotemporal control of STML lasers is not always needed, and some simple yet robust control can be valuable for applications. As an example, a simple fiber shaper has been used for external control of ultrashort pulse mode content and supercontinuum generation in a MMF recently, which proves useful for nonlinear microscopy^197^.

In summary, we have introduced the fundamentals of STML in MMF lasers in the review. We also summarize the progress of STDS spatiotemporal dynamics and its measurement. As a new type of nonlinear system and ultrafast science testbed, STML fiber lasers have extended the landscape of dissipative soliton physics and are gaining increasing interest. We believe more exotic spatiotemporal phenomena can be observed and harnessed to build better ultrafast lasers with unprecedented spatial complexity. All these developments can help to reveal light behavior and physics in loosely confined waveguides with intermediate nonlinearity. In practice, STDS with engineered spatial mode from MMF laser may be used in micromachining^68^, nonlinear microscopy^71,72,197^ and optical tweezing^70^.
